# Integrated neural network framework for multi-object detection and recognition using UAV imagery

**DOI:** 10.3389/fnbot.2025.1643011

**Published:** 2025-07-30

**Authors:** Mohammed Alshehri, Tingting Xue, Ghulam Mujtaba, Yahya AlQahtani, Nouf Abdullah Almujally, Ahmad Jalal, Hui Liu

**Affiliations:** ^1^Department of Computer Science, King Khalid University, Abha, Saudi Arabia; ^2^School of Environmental Science & Engineering, Nanjing University of Information Science and technology, Nanjing, China; ^3^Cognitive Systems Lab, University of Bremen, Bremen, Germany; ^4^Department of Computer Science, Air University, Islamabad, Pakistan; ^5^Department of Informatics and Computer Systems, King Khalid University, Abha, Saudi Arabia; ^6^Department of Information Systems, College of Computer and Information Sciences, Princess Nourah bint Abdulrahman University, Riyadh, Saudi Arabia; ^7^Department of Computer Science and Engineering, College of Informatics, Korea University, Seoul, Republic of Korea; ^8^Guodian Nanjing Automation Co., Ltd, Nanjing, China; ^9^Jiangsu Key Laboratory of Intelligent Medical Image Computing, School of Artificial Intelligence, Nanjing University of Information Science and Technology, Nanjing, China

**Keywords:** Unmanned Aerial Vehicle, neural network models, deep learning, multi-object recognition, transfer learning, intelligent detector, autonomous system

## Abstract

**Introduction:**

Accurate vehicle analysis from aerial imagery has become increasingly vital for emerging technologies and public service applications such as intelligent traffic management, urban planning, autonomous navigation, and military surveillance. However, analyzing UAV-captured video poses several inherent challenges, such as the small size of target vehicles, occlusions, cluttered urban backgrounds, motion blur, and fluctuating lighting conditions which hinder the accuracy and consistency of conventional perception systems. To address these complexities, our research proposes a fully end-to-end deep learning–driven perception pipeline specifically optimized for UAV-based traffic monitoring. The proposed framwork integrates multiple advanced modules: RetinexNet for preprocessing, segmentation using HRNet to preserve high-resolution semantic information, and vehicle detection using the YOLOv11 framework. Deep SORT is employed for efficient vehicle tracking, while CSRNet facilitates high-density vehicle counting. LSTM networks are integrated to predict vehicle trajectories based on temporal patterns, and a combination of DenseNet and SuperPoint is utilized for robust feature extraction. Finally, classification is performed using Vision Transformers (ViTs), leveraging attention mechanisms to ensure accurate recognition across diverse categories. The modular yet unified architecture is designed to handle spatiotemporal dynamics, making it suitable for real-time deployment in diverse UAV platforms.

**Method:**

The framework suggests using today’s best neural networks that are made to solve different problems in aerial vehicle analysis. RetinexNet is used in preprocessing to make the lighting of each input frame consistent. Using HRNet for semantic segmentation allows for accurate splitting between vehicles and their surroundings. YOLOv11 provides high precision and quick vehicle detection and Deep SORT allows reliable tracking without losing track of individual cars. CSRNet are used for vehicle counting that is unaffected by obstacles or traffic jams. LSTM models capture how a car moves in time to forecast future positions. Combining DenseNet and SuperPoint embeddings that were improved with an AutoEncoder is done during feature extraction. In the end, using an attention function, Vision Transformer-based models classify vehicles seen from above. Every part of the system is developed and included to give the improved performance when the UAV is being used in real life.

**Results:**

Our proposed framework significantly improves the accuracy, reliability, and efficiency of vehicle analysis from UAV imagery. Our pipeline was rigorously evaluated on two famous datasets, AU-AIR and Roundabout. On the AU-AIR dataset, the system achieved a detection accuracy of 97.8%, a tracking accuracy of 96.5%, and a classification accuracy of 98.4%. Similarly, on the Roundabout dataset, it reached 96.9% detection accuracy, 94.4% tracking accuracy, and 97.7% classification accuracy. These results surpass previous benchmarks, demonstrating the system’s robust performance across diverse aerial traffic scenarios. The integration of advanced models, YOLOv11 for detection, HRNet for segmentation, Deep SORT for tracking, CSRNet for counting, LSTM for trajectory prediction, and Vision Transformers for classification enables the framework to maintain high accuracy even under challenging conditions like occlusion, variable lighting, and scale variations.

**Discussion:**

The outcomes show that the chosen deep learning system is powerful enough to deal with the challenges of aerial vehicle analysis and gives reliable and precise results in all the aforementioned tasks. Combining several advanced models ensures that the system works smoothly even when dealing with problems like people being covered up and varying sizes.

## 1 Introduction

Robotic perception has transformed greatly because of neural network–based algorithms and deep learning models that can learn real-world data, adjust to different situations, and decide intelligently. Technological progress has allowed robots to adjust their automation and intelligence in many different environments (Hanzla and Jalal, 2025). In the field of UAVs, it is tough for perception systems because the environment is very changeable, featuring occlusions, distorted views, fast movement, changing sizes, and uneven lighting (Mohammed et al., 2025). Since these factors are important, technologies must be efficient, reliable, and scalable for rapid handling of scene comprehension and instant decisions. Tools such as Unmanned Aerial Vehicles are now important for services like traffic management, disaster aid, security, and protecting the environment (Hossain et al., 2019; Ghulam et al., 2024). They need to be able to correctly interpret what takes place from above. On the other hand, when there is noise, dynamic scenes or many objects close together, old methods of computer vision have difficulty understanding the images (Mujtaba et al., 2025). For this reason, DNNs are now widely used because they can handle complicated feature extraction and work well in various scenarios. While many deep learning models work well on individual issues such as locating or tracking objects, there are not many that help do several tasks together as a complete system in aerial scenarios (Bisma et al., 2025). Many existing solutions do not change easily, are not able to grow large or provide inconsistent outcomes in the real world. Therefore, this research introduces a neural network-centered process especially fitted for analyzing aerial vehicles (Mohammed et al., 2025). Detection, tracking, counting, trajectory prediction, and classification are combined through deep learning framework into one operational framework (Waqas et al., 2025a,b). Every element is picked or built to handle certain issues in aerial imagery, providing high accuracy, strong performance, and easy processing in real-time. The conceptual advance of this work lies in the integration of diverse neural architectures into a single unified pipeline that leverages their complementary strengths (Chughtai, 2023a,b). RetinexNet enhances visibility under poor lighting conditions. HRNet performs high-resolution semantic segmentation for precise object localization. YOLOv11 delivers fast and accurate vehicle detection (Mujtaba et al., 2025). Deep SORT incorporates convolutional appearance features and motion prediction for robust tracking. CSRNet is utilized for density map-based vehicle counting, while LSTM models capture temporal dependencies for accurate trajectory prediction (Mahwish and Ahmad, 2023). To create the classifier, the features are improved by mixing DenseNet and SuperPoint and AutoEncoder is used to refine them. In the end, a Vision Transformer uses attention to both improve performance and make results easier to interpret. The primary objective of this study is to develop a unified, end-to-end deep learning framework for UAV-based vehicle perception that integrates multiple neural models to perform image enhancement, detection, tracking, counting, trajectory prediction, and classification in real-world aerial environments.

The key contributions of this work are as follows

- Unified End-to-End Neural Architecture: This study presents a fully integrated deep learning–based perception pipeline for UAV traffic monitoring. Each module in the system ranging from image enhancement and semantic segmentation to detection, tracking, counting, trajectory prediction, and vehicle classification is individually optimized using state-of-the-art neural models tailored for aerial vehicle surveillance.
- Seamless Spatiotemporal Integration: The architecture is designed to allow robust interconnection between neural modules, enabling the efficient fusion of spatial and temporal information. This design significantly improves system coherence, adaptability, and reliability in dynamic aerial environments.
- Robust Performance: Our proposed framework demonstrates outstanding generalizability and accuracy when evaluated on two benchmark datasets which is AU-AIR and Roundabout. It effectively handles occlusions, lighting variations, and multi-scale object scenarios, confirming its practical applicability for aerial traffic monitoring and autonomous systems in Real word.
- Cross-Platform Versatility and Scalability: Designed for deployment across various UAV platforms, the proposed system shows strong adaptability to diverse urban and semi-urban conditions. This makes it suitable for widespread implementation in intelligent transportation systems, surveillance operations, and many military operations.

The system is tested on two benchmark datasets, AU-AIR and Roundabout, which are known to be very difficult for aerial traffic analysis. This is shown by an excellent accuracy rate, as well as generalization skills, as the pipeline achieves better results than other methods in all three areas.

## 2 Literature review

In recent years. UAVs have gained significant traction because of their applications in traffic monitoring and urban planning. However, many challenges like occlusion, scale variation, and complex environments demand robust and efficient deep learning solutions. In recent research, researchers explored advanced architectures including a convolutional and transformer-based model for detection, tracking, and classification the following literature review highlights key developments in these domain.

### 2.1 Vehicle detection and tracking systems

Accurate vehicle detection and tracking in aerial imagery is a critical task for enabling intelligent transportation systems, urban traffic analysis, and autonomous navigation. The unique constraints imposed by UAV-captured data including small object sizes, occlusions, variable illumination, and motion-induced artifacts have driven extensive research in this area. Several prior studies have attempted to tackle these challenges using a range of classical and deep learning-based approaches. In a recent study, Bouguettaya et al. (2021) present a comprehensive review of deep learning techniques for vehicle detection in UAV imagery, highlighting both the opportunities and limitations of current methods. Their work systematically categorizes deep architectures such as CNNs, RNNs, Autoencoders, and GANs and discusses their suitability for aerial perspectives where traditional handcrafted features struggle. Importantly, they point out that shallow learning approaches often fall short in generalizing across complex UAV scenarios, thereby reinforcing the relevance of deep learning framework. This directly aligns with our pipeline’s adoption of advanced architectures like YOLOv11, HRNet, and Vision Transformers, which were chosen precisely for their ability to generalize across diverse and noisy aerial environments. In another study, Yu et al. (2020) take a complementary approach by introducing a high-quality UAV dataset specifically designed to challenge conventional object detection and tracking algorithms. Their dataset features high-density traffic, fast camera motion, and small object scale characteristics that mirror the real-world complexities tackled in our experiments using the AU-AIR and Roundabout datasets. Moreover, their proposed Context-aware Multi-task Siamese Network (CMSN) integrates contextual cues to improve tracking robustness, a concept that resonates with our fusion of spatio-temporal modeling through LSTM and feature-level enhancements using SuperPoint and DenseNet (Bisma and Ahmad, 2023a). Their study confirms that effective performance in UAV contexts often requires an ensemble of modules capable of reasoning across frames and features an approach we fully adopt in our unified pipeline.

Another author, Singh et al. (2022) present a hybrid intelligent framework combining classical image processing with modern machine learning for vehicle detection, tracking, and geolocation in UAV imagery. While their methodology includes adaptive filtering, morphological transformations, and clustering-based motion analysis, they also integrate a Fast-RCNN module to refine detection. Although their architectural choices differ from ours focusing more on lightweight classical pipelines their recognition of real-time constraints and the need for robustness in dynamic traffic scenes strongly complements our goal of building an end-to-end, real-time UAV system. Their emphasis on practical deployment, noise handling, and multi-step reasoning is particularly relevant to our motivation for using modules like Deep SORT for tracking and CSRNet for vehicle density estimation (Mujtaba and Ahmad, 2024). Collectively, these works establish a strong theoretical and empirical foundation that motivates the need for a comprehensive, modular, and adaptable vehicle analysis framework. However, while each prior study addresses specific sub-tasks such as detection, tracking, or trajectory prediction our research advances the field by integrating all core functionalities into a single deep learning-driven pipeline.

### 2.2 Vehicle detection and classification systems

Accurate vehicle detection and classification underpin many UAV applications such as traffic management, parking supervision, and intelligent transportation systems. The small size of vehicles in aerial images, complex backgrounds, and the presence of visually similar objects pose persistent challenges to traditional algorithms. In a recent study, Kumar et al. (2022) addressed these issues by proposing a CNN-based vehicle detection and classification algorithm capable of distinguishing between light and heavy vehicles. Their method, validated on multiple aerial image datasets like VEDAI and VIVID, achieved high accuracy and robustness across varied scenarios. This work supports the need for specialized classification modules that handle vehicle diversity and complex visual context elements embedded in our DenseNet and Vision Transformer classification components. Similarly, Lin et al. (2020) introduced the VAID dataset, a well-annotated aerial image collection designed for training and evaluating vehicle detection algorithms under diverse traffic conditions. Their experiments demonstrated that domain-specific datasets significantly improve detection accuracy, a principle we have adopted by utilizing datasets such as AU-AIR and Roundabout to ensure model generalization in real-world UAV environments. Berwo et al. (2023) provide a recent, extensive review of DL-based vehicle detection and classification techniques relevant to Intelligent Transportation Systems. Their overall survey emphasizes advances in network architectures, benchmark datasets, and real-time applications including toll management and traffic density estimation. They identify challenges in appearance-based recognition and highlight the growing demand for robust, scalable, and efficient deep learning solutions, corroborating the rationale behind our adoption of YOLOv11, HRNet, and Vision Transformers for the detection and classification stages (Shuja and Ahmad, 2023). By synthesizing insights from the above works, we build upon state-of-the-art methodologies, tailoring deep learning architectures and leveraging rich datasets to address the multifaceted challenges of aerial vehicle analysis. In contrast to existing models that often struggle with real-time adaptability, our pipeline is designed to sustain high precision across diverse environments by integrating temporal consistency and attention-based reasoning. The incorporation of Deep SORT enhances tracking stability, while CSRNet ensures the precise vehicle counting even in congested scenes. The use of LSTM and Vision Transformers ensure accurate vehicle classifications. This makes our system responsive under varying lighting conditions, motion blur, and occlusions commonly encountered in UAV operations.

## 3 Pipeline design and implementation

### 3.1 Proposed methodology

**Figure 1:**
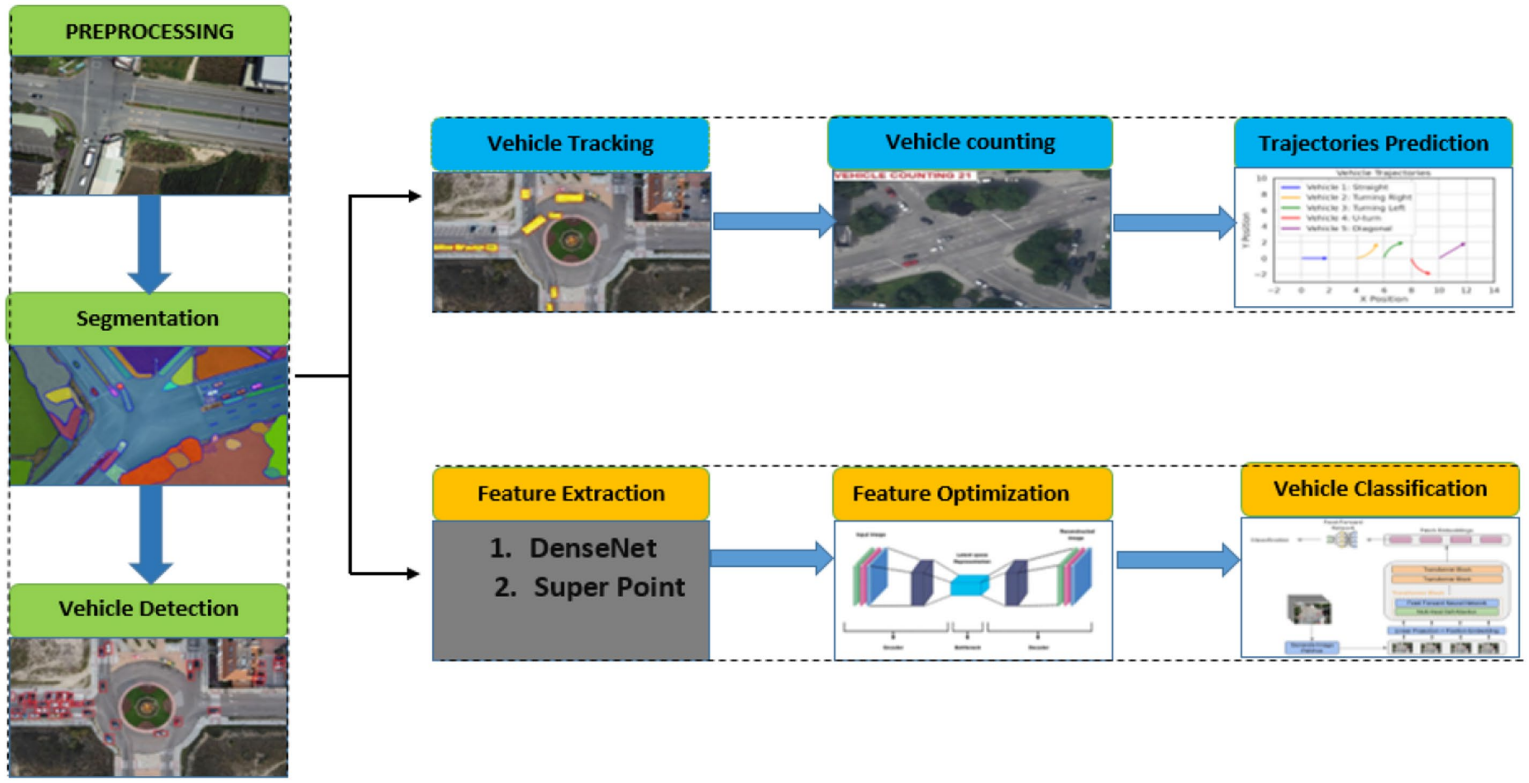
Overview of the proposed deep learning–based aerial vehicle analysis system.

The core conceptual advance of this work is the design of a unified, end-to-end deep learning based framework that integrates diverse deep learning models each tailored to a specific perception task into a coherent system optimized for aerial vehicle analysis. As shown in Figure 1, the pipeline begins with image enhancement using RetinexNet to correct illumination and recover details in aerial frames captured under suboptimal lighting (Ghulam and Ahmad, 2024). This is followed by HRNet, which performs high-resolution semantic segmentation to preserve spatial precision in object boundaries. YOLOv11 is then employed for rapid and accurate vehicle detection, after which Deep SORT ensures robust tracking. CSRNet is utilized to estimate object density for precise vehicle counting, even in densely populated scenes (Azmat and Ahmad, 2021). To capture temporal dynamics for trajectory prediction, LSTM networks model motion patterns across consecutive frames. Rich spatial features are extracted using DenseNet and SuperPoint, and subsequently refined via an AutoEncoder to enhance compactness and discriminability. The final classification is handled by a Vision Transformer (ViT), which applies attention-based modeling to improve classification accuracy and interpretability (Naseer and Jalal, 2025a,b). This interconnected pipeline enables seamless spatiotemporal integration by allowing spatial information from HRNet, YOLOv11, and CSRNet to be temporally correlated using Deep SORT and LSTM modules. Each module communicates through shared feature maps and object identities, enabling consistent understanding of vehicle behavior across both space and time. The proposed methodology is fully modular, scalable, and entirely driven by neural networks, offering a robust and efficient solution for aerial robotic perception in complex, real-world environments.

### 3.2 Pre-processing of dataset via RetinexNet

The preprocessing stage supports the entire pipeline by making sure the images from UAVs are fit for use in training deep-learning tools (Waqas et al., 2025a,b). Images taken from the high altitude are easily affected by varying amounts of light, shadows and a difference between bright and dark spots. We therefore use RetinexNet, a model trained with machine learning that applies the Retinex concept to split an image into its reflectance and lighting portions (Hai et al., 2023). RetinexNet was selected due to its ability to enhance contrast and visibility in UAV images captured under uneven or low lighting conditions, which are common in aerial surveillance (Ahmed et al., 2024; Muneeb et al., 2023). The data is preprocessed in advance by making all frames the same size, 512 × 512 pixels and changing the pixel values to fall within the range 0 to 1. The training set is processed using random cropping, horizontal flipping and changes in brightness to help the model perform better in many scenarios. This method helps the framwork discover features that do not depend on the light source which is essential for using the system in different types of light (Mahammed et al., 2023). RetinexNet operates through a decomposition-and-enhancement mechanism, formally defined in Equation 1:


(1) I(x,y)=R(x,y).L(x,y)

Here, I(x,y) represents the observed aerial image at pixel location (x,y)=R(x,y) is the reflectance component containing structural and color information, and L(x,y) denotes the illumination map. The network first learns to estimate L(x,y ) using a Decomposition Network (Decom-Net), after which an Enhancement Network (Enhance-Net) adjusts illumination while preserving reflectance. The loss function guiding decomposition is a combination of structure-preserving and smoothness constraints as presents in Equation 2:


(2) $$L_{\text{decom}}=I-R.L_{1}+\lambda_{1}\nabla L{\left\|\;1+\lambda_{2}R-1\right\|}_{1}$$

where ∥·∥_1_ denotes the L1 norm, ∇L represents the spatial gradient of the illumination map enforcing smoothness, and λ_1_, λ_2_ are weighting factors controlling the balance between fidelity and regularization. To further improve feature visibility under harsh lighting, we define a contrast enhancement objective. This process is mathematically defined in Equation 3:


(3) $$L_{\mathrm{enh}}=\sum_{x,y}[{\left(R(x,y)-\mathrm{\mu R}\;\right)}^{2}]$$

where μR is the mean reflectance across the image, promoting contrast maximization. The enhanced images produced from this step serve as higher-quality inputs for segmentation and detection, effectively reducing errors caused by low-visibility regions and enabling the neural components of the pipeline to operate under more consistent and informative visual conditions. This process is defined in Equation 1.

### 3.3 Segmentation via high-resolution network

We integrate a high-resolution semantic segmentation mechanism that preserves spatial fidelity in aerial imagery, enabling precise foreground-background separation critical for downstream tasks. Traditional segmentation models often suffer from spatial degradation due to repeated pooling and downsampling operations, which are especially detrimental when processing aerial scenes where object boundaries are small and closely packed (Wang et al., 2017; Ahmad et al., 2021). To address this, we incorporate the High-Resolution Network (HRNet) a deep convolutional neural architecture that maintains high-resolution representations throughout the entire forward pass (Naseer et al., 2024). HRNet maintains high-resolution features throughout the network, making it suitable for precise vehicle segmentation in UAV imagery, especially for small and overlapping objects. HRNet processes aerial frames enhanced by RetinexNet and outputs dense semantic masks, classifying each pixel as either vehicle or background with fine-grained accuracy (Xie et al., 2020). The model achieves this by concurrently executing multiple convolutional branches at different resolutions and continuously exchanging information across them, allowing it to learn both global context and local structural details in Equation 4 (Naseer and Jalal, 2024). The segmentation task is mathematically defined in Equation 4 as a pixel-wise classification problem, optimized through a composite loss function. The categorical cross-entropy loss guides the primary objective:


(4) $$L_{\mathrm{tv}}=\sum_{x,y}\;\sum_{c=1}^{C}Y_{c}\;(x,y)\log P_{c}(x,y)$$

where $Y_{c}\;(x,y)$ is the ground truth label for class ccc at pixel location $\left(x,y)\log P_{c}(x,y\right)$ is the predicted class probability, and C is the number of segmentation categories. To reinforce spatial smoothness and mitigate prediction noise near object boundaries, we include a total variation loss as presents in Equation 5:


(5) $$L_{\mathrm{tv}}=\sum_{x,y}\;\;(|\nabla_{x}P\;(x,y)|+\|\nabla_{y}\;P(x,y)\|)$$

**Figure 2:**
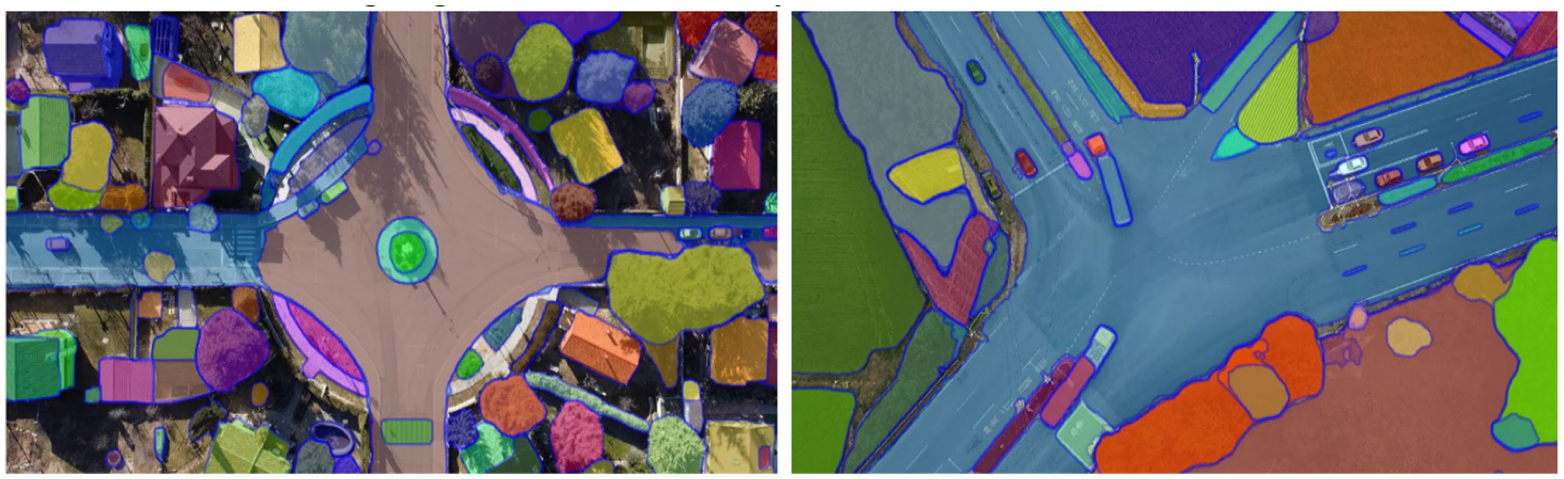
Output masks generated by HRNet demonstrating fine-grained vehicle segmentation.

**Figure 3:**
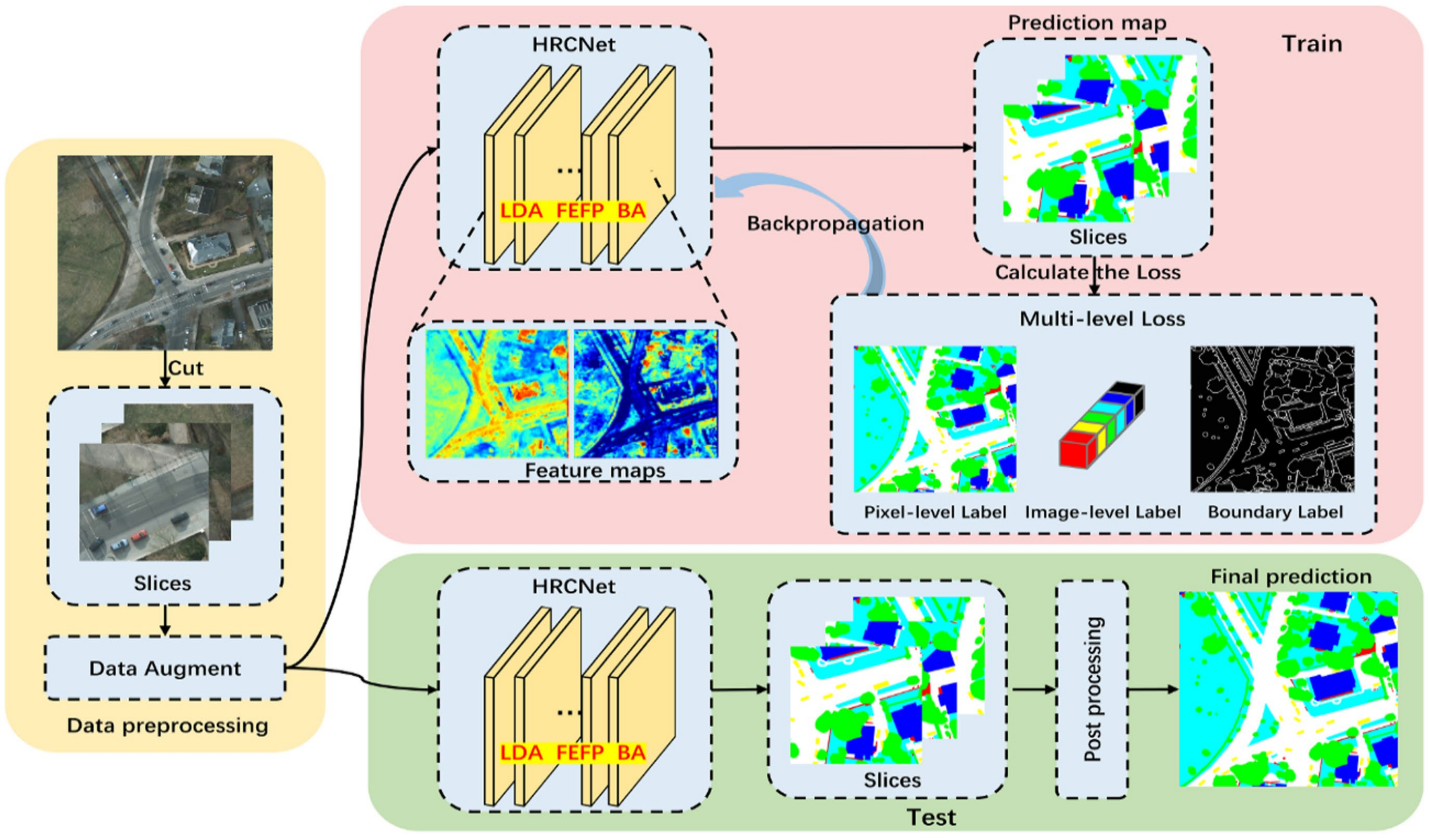
Overview of the HRNet-based semantic segmentation architecture.

This term penalizes sharp transitions in adjacent pixels, encouraging the network to produce coherent object masks. Additionally, we implement a boundary alignment loss to improve edge precision as shown in Equation 6:


(6) $$L_{\text{edge}}=\sum_{x,y}|\nabla P\;(x,y)-\nabla Y(x,y){|}^{2}$$

where ∇P(x,y) and ∇Y(x,y) represent the gradient maps of the predicted and ground truth masks, respectively. The final segmentation loss is expressed as $L_{\text{total}}=L_{\mathrm{seg}}+\alpha L_{\mathrm{tv}}+BL_{\text{edge}}$ with *α* and *β* controlling the regularization strength (Chughtai, 2023a,b). The results of the segmentation module are illustrated in Figure 2, while a comprehensive overview of the high-resolution segmentation framework is depicted in Figure 3. The figure outlines the complete HRNet-based pipeline, including data preprocessing, training, and inference stages (Abrar et al., 2019). During training, multi-level supervision is employed through pixel-wise, image-level, and boundary-level loss components, all of which contribute to enhancing segmentation accuracy and robustness.

### 3.4 YOLOv11-based vehicle detection

The core conceptual advance at this stage lies in the integration of an ultra-fast and accurate object detection framework YOLOv11, within the aerial vehicle analysis pipeline (He et al., 2025). Designed to deliver improved performance without compromising detection precision, YOLOv11 addresses key challenges posed by aerial imagery, including small object scales, varied orientations, and dense scene layouts (Hanzla et al., 2024a). YOLOv11 was chosen for its balance between detection accuracy and incredible performance, particularly for detecting small, fast-moving objects from aerial views. Unlike traditional region proposal-based detectors that are computationally intensive, YOLOv11 employs a single-stage, fully convolutional architecture that directly predicts bounding boxes and class probabilities from input images, enabling efficient inference on UAV-captured data streams. In the context of the proposed pipeline, YOLOv11 processes the segmentation-refined frames and identifies vehicle instances across diverse spatial configurations, providing precise bounding box coordinates for downstream tracking and counting operations (Ayesha and Ahmad, 2021). YOLOv11 extends the foundational YOLO architecture through multiple enhancements (Chaman et al., 2025). It integrates Cross-Stage Partial (CSP) connections to improve gradient flow and reduce computational complexity, Spatial Pyramid Pooling Fast (SPPF) for robust multi-scale feature aggregation, and an improved anchor-free detection head to better localize small vehicles in aerial views. The input to YOLOv11 is a high-resolution image where non-vehicle regions have been suppressed via segmentation, allowing the network to focus computational attention on relevant areas (Waheed et al., 2023). The detection head generates a fixed grid of anchor points, as illustrated in Equation 7 each predicting object presence and bounding box adjustments, optimized using the Complete IoU (CIoU) loss:


(7) $$\text{LCIou}=1-\mathrm{IoU}+\frac{P^{2}(b.b^{\mathrm{gt}})}{C^{2}}+\mathrm{\alpha v}$$

**Figure 4:**
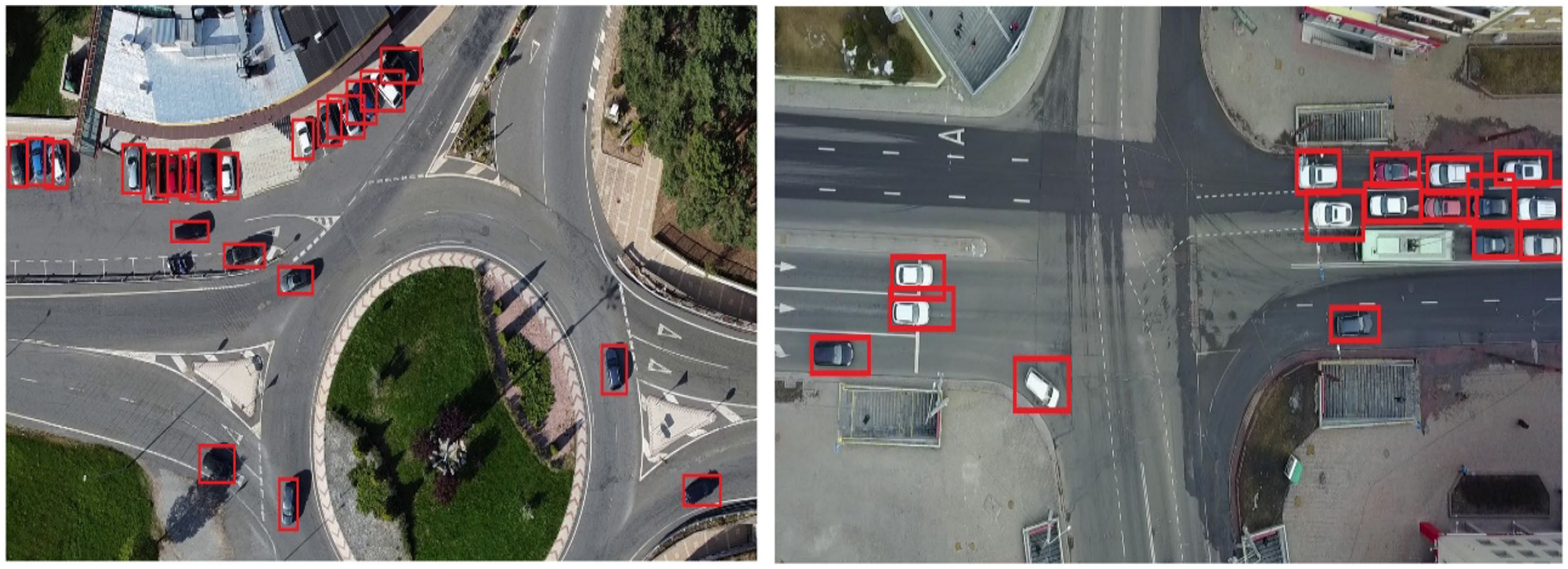
The output of YOLOv11 demonstrating accurate detection across complex aerial scenes.

**Figure 5:**
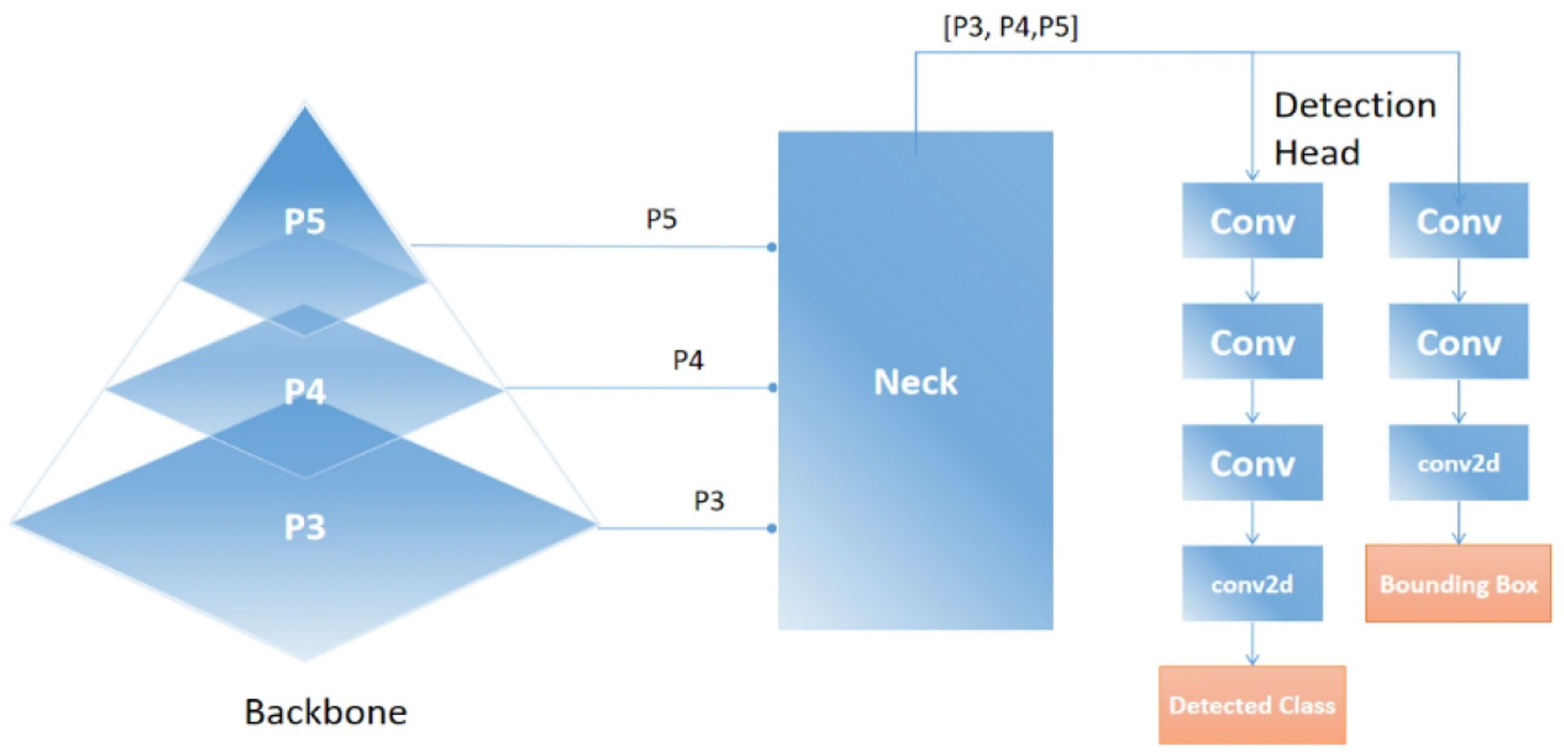
Overview of the YOLOv11 detection framework, including backbone, neck, and head.

Here, IoU is the intersection-over-union between the predicted box b and ground truth $b^{\mathrm{gt}}$ *P* denotes the Euclidean distance between their center points, c is the diagonal length of the smallest enclosing box, and v captures aspect ratio consistency. The term α balances the influence of shape alignment, resulting in more stable convergence. To improve classification robustness, YOLOv11 incorporates Focal Loss. This complete CIoU formulation is defined in Equation 8:


(8) $$L_{\mathrm{cls}}=-\sum_{i=1}^{N}\mathrm{\alpha t}(1-P_{t})\log(\mathrm{pt})$$

where *pt* is the predicted confidence for the true class label, α_t_ is a class-specific weighting factor, and *γ* modulates the down-weighting of easy examples. This formulation mitigates class imbalance by focusing learning on hard-to-detect vehicles, especially in cluttered aerial scenes. The main conceptual advance in this stage is the integration of YOLOv11, which delivers real-time, high-precision vehicle detection in complex aerial imagery (Chughtai and Jalal, 2024). By applying Non-Maximum Suppression (NMS), the model eliminates redundant predictions and outputs refined bounding boxes with class labels and confidence scores. As illustrated in Figure 4, YOLOv11 accurately detects vehicles across varying scales, orientations, and densities. Figure 5 provides a high-level architectural overview of YOLOv11, highlighting the flow of multi-scale feature maps through the backbone, neck, and detection head. This structure enables the model to robustly detect objects at different resolutions by fusing spatial and semantic information effectively. The integration of the YOLOv11 in our proposed pipeline marks a pivotal enhancement, addressing key UAV-specific challenges such as occlusion, very dense environment, and scale variations. YOLOv11 architectural innovations enable high recall and precision, especially in very complex aerial scenes. This very robust detection capability serves as the backbone of subsequent tracking and counting modules. YOLOv11 is not merely a detection module but it acts as a critical enabler of accurate, scalable, and timely aerial vehicle analysis in the proposed end-to-end pipeline.

### 3.5 Robust vehicle tracking in aerial imagery using deep SORT integration

The principal conceptual advance introduced at this stage is the deployment of Deep SORT (Simple Online and Real-time Tracking with a Deep Association Metric), which enables robust, vehicle tracking with sustained identity preservation across video frames (Hou et al., 2019). Deep SORT was selected for its ability to maintain identity consistency over frames, even under occlusion or rapid movement, which is essential for stable tracking in UAV footage (Alonazi et al., 2023; Raza et al., 2023). In the context of aerial vehicle analysis, Deep SORT effectively addresses challenges such as occlusion, abrupt motion, and appearance variations, ensuring consistent tracking of vehicles over time in dynamic environments. Deep SORT builds upon traditional SORT by incorporating appearance features extracted via a Convolutional Neural Network (CNN), which are used alongside Kalman filtering for motion estimation and the Hungarian algorithm for optimal data association (Mahwish et al., 2021). The tracking process begins by feeding the bounding boxes and detection confidences from YOLOv11 into the Deep SORT tracker. Each detected vehicle initializes a new track or updates an existing one, depending on how well it matches existing trajectories (Raza et al., 2023). The Kalman filter predicts each object’s future location based on a linear motion model. The predicted bounding boxes are then compared with new detections using two metrics: Mahalanobis distance for motion similarity and cosine similarity for appearance affinity. The Mahalanobis distance between a detection *d* and a predicted track t shown in Equation 9:


(9) $$\mathrm{DM}(d,t)=\sqrt{{\left(d-t\right)}^{T}(S^{-1}(d-t)}$$

where *t* is the predicted state from the Kalman filter and *S* is the covariance matrix of the prediction. This metric ensures that only spatially plausible matches are considered, reducing erroneous associations for appearance matching, each detection is embedded into a high-dimensional feature space using a pre-trained CNN. The cosine similarity between the embedding vectors $e_{i}$ and $e_{j}$ of a track and detection, respectively, is computed in Equation 10:


(10) $$\mathrm{Si}m_{\cos}(e_{i},e_{i})=,\frac{e_{i}.e_{j}}{\left\|e_{i}\|\|e_{j}\right\|}$$

**Figure 6:**
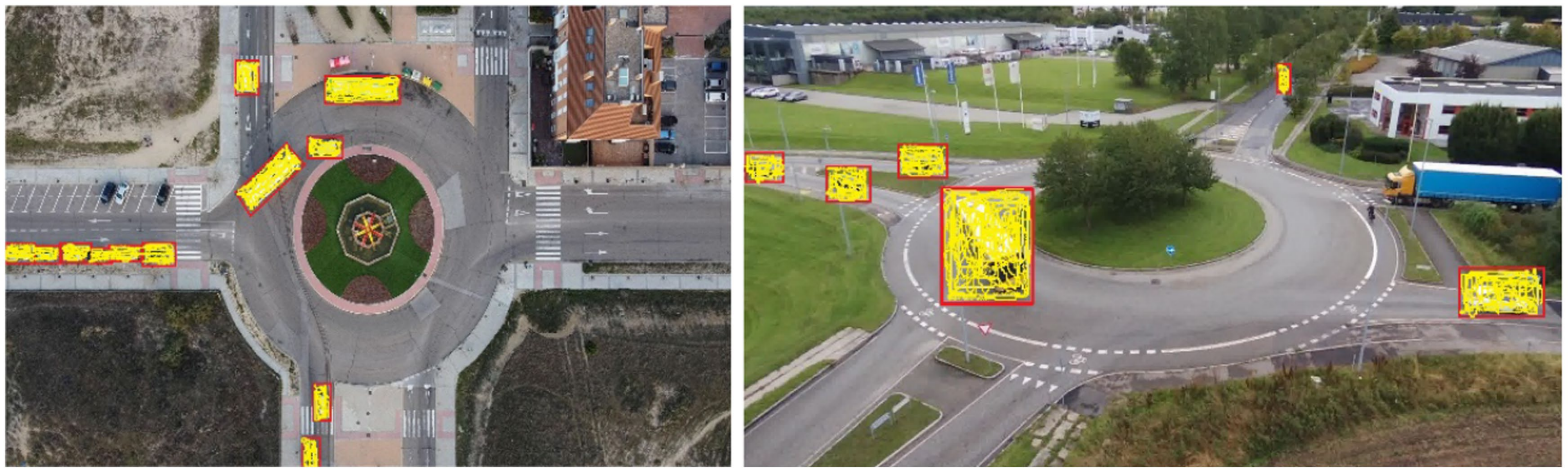
Visualization of Deep SORT tracking.

A higher cosine similarity indicates a stronger visual match. These spatial and appearance affinities are jointly used to construct a cost matrix for the Hungarian algorithm, which then assigns detections to existing tracks in a globally optimal way. As illustrated in Figure 6, Deep SORT successfully maintains unique identities for each vehicle across multiple frames, even in dense traffic and occlusion-prone scenarios. This enables the system to extract continuous trajectories and provides reliable temporal context for downstream tasks such as counting and trajectory prediction.

### 3.6 Vehicle counting using CSRNet

The key conceptual advancement in the vehicle counting phase lies in leveraging CSRNet, a deep convolutional neural network specifically designed for accurate crowd density estimation, adapted here for precise vehicle counting in aerial imagery. CSRNet performs well in dense and complex traffic scenes without requiring precise bounding boxes, making it well-suited for aerial vehicle counting where detection overlap is high (Pervaiz et al., 2023). Unlike traditional counting approaches that depend solely on discrete object detections, CSRNet generates continuous density maps that capture both visible and partially occluded vehicles, effectively handling challenges such as overlapping objects, scale variation, and perspective distortion inherent in UAV-captured scenes (Guo et al., 2022). After vehicle detection with YOLOv11, the aerial images either the original frames or refined by detected bounding boxes serve as input to CSRNet. CSRNet employs dilated convolution layers to enlarge the receptive field while preserving spatial resolution, enabling the network to aggregate multi-scale contextual information essential for reliable density estimation (Mahwish et al., 2021). This process produces a density map *D(x,y)* where each pixel’s value reflects the estimated vehicle density at that location. CSRNet maps an input image *I* to a density estimate via a nonlinear function $f_{0}$ parameterized by network weights *θ*. This mapping is formally defined in Equation 11:


(11) $$D(x,y)=f_{0}\;(I)$$

where $f_{0}\;$ denotes the CSRNet model parameterized by weights θ. The total vehicle count C is obtained by integrating the density map over the image domain *Ω*. This integration is defined in Equation 12:


(12) C=∫ΩD(x,y)dx,dy

**Figure 7:**
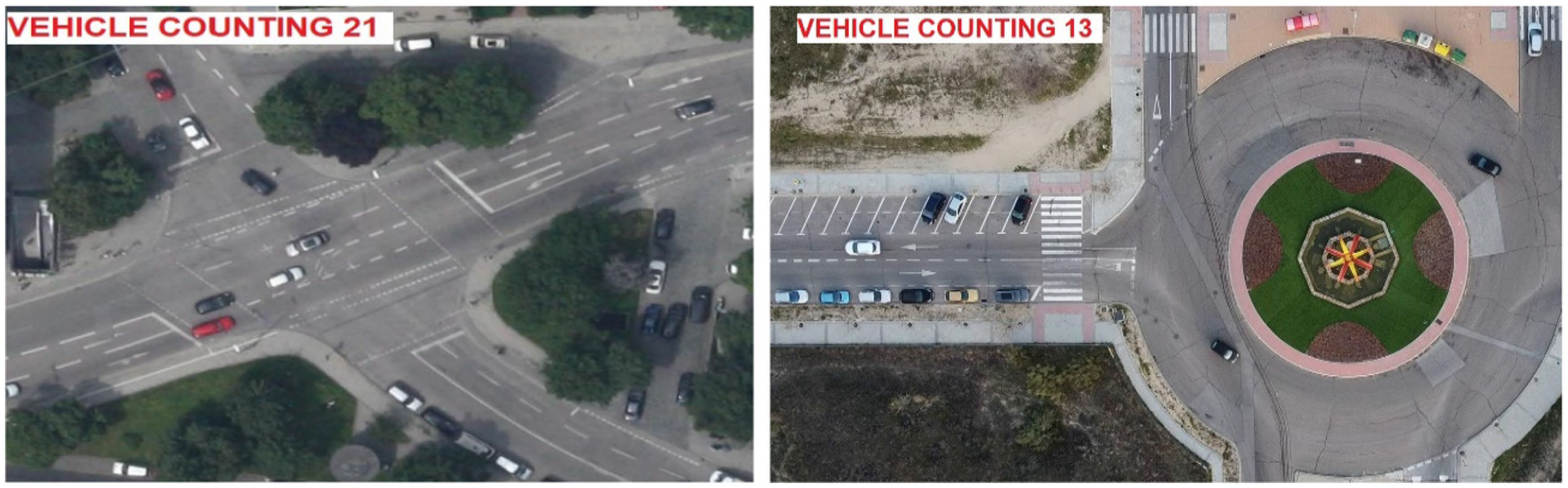
Output of CSRNet demonstrating precise vehicle count estimation in UAV imagery.

During training, CSRNet minimizes the mean squared error (MSE) between the estimated density map D and the ground truth density map D. This loss function is given in Equation 13:


(13) $$L(0)=\frac{1}{N}\sum_{i=1}^{N}\|D_{i}-D_{i}\;\|\frac{2}{2}$$

where N is the number of training samples. Integrating CSRNet within the proposed pipeline allows for robust, scalable vehicle counting that complements YOLOv11’s bounding box detection by capturing the spatial distribution of vehicles comprehensively even under heavy occlusion or congested traffic scenarios. The use of this approach improves the pipeline’s capability to give consistent information about the traffic flow required for smart traffic monitoring and perfect navigation for robots. The vehicle counting is shown in Figure 7 and proves that CSRNet shows high precision and reliability under difficult image conditions with various traffic sizes.

### 3.7 Vehicle trajectory prediction using LSTM networks

**Figure 8:**
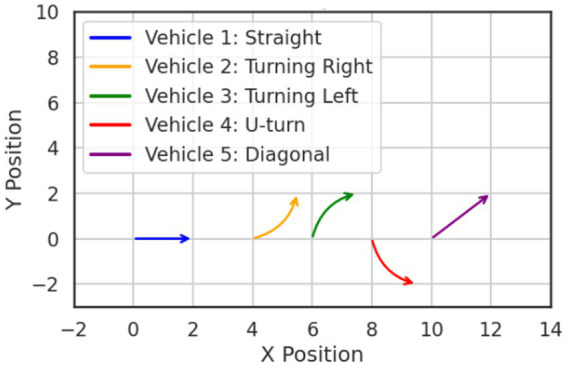
Visualization of temporal vehicle movement prediction using LSTM.

The trajectory prediction phase introduces a significant conceptual advancement by incorporating Long Short-Term Memory (LSTM) networks to model and forecast the temporal dynamics of vehicle motion in aerial surveillance (Altché and de La Fortelle, 2017). LSTM was used due to its strength in capturing temporal dependencies across sequential data, allowing it to model complex vehicle movement patterns in continuous aerial video. Unlike conventional motion models that rely on linear or rule-based assumptions, LSTMs are designed to capture long-range dependencies and nonlinear temporal patterns from sequential data, making them computationally effective for learning motion behaviors in dynamic, unconstrained environments (Hafeez et al., 2021). In this stage, the vehicle trajectories are generated based on the outputs of the Deep SORT tracking module. Specifically, each vehicle’s bounding box center coordinates $x_{t},y_{t}$ are extracted over consecutive time steps to form a sequence $S=(x_{1},y_{1}),(x_{2},y_{2}),\ldots .(x_{t},y_{t})$ where T denotes the number of past frames. This sequence is used as the input to the LSTM, which learns to map the historical motion patterns to future positional estimates. An LSTM unit consists of a memory cell $C_{t}$ a hidden state $h_{t}$ and three gates: input $i_{t}$ , forget $f_{t}$ , and output $O_{t}$ , which control the flow of information. At each time step *t*, the LSTM performs the following updates:


(14) $$f_{t}=\sigma (W_{f}.[h_{t-1,}x_{t}]+b_{f})$$

(15) $$i_{t}=\sigma (W_{i}.[h_{t-1,}x_{t}]+b_{i})$$

(16) $$C_{t}=\tanh(\mathrm{Wc}.[h_{t-1,}x_{t}]+b_{c})$$

(17) $$C_{t}=f_{t}⊙C_{t-1}+i_{t}⊙C_{t}$$

(18) $$O_{t}=\sigma (W_{o}.[h_{t-1,}x_{t}]+b_{o})$$

(19) $$h_{t}=O_{t}⊙\tanh(C_{t})$$

where *σ* denotes the sigmoid activation function, tanh is the hyperbolic tangent, and ⊙ indicates element-wise multiplication. The matrices *W* and vector *b* are learnable parameters. Equations 14–19 represent the full internal operation of the LSTM cell, from memory update to output generation, forming the mathematical backbone of the trajectory prediction process. The output of the LSTM at the final time step is passed through a dense layer to generate predicted coordinates $X_{t+1,}YT_{+t}$ extending the vehicle’s path beyond the observed time window. This enables the system to anticipate future positions even in complex traffic conditions, facilitating higher-level decision-making and interaction modeling for autonomous systems. Figure 8 illustrates a representative graph of the predicted vehicle trajectory over time, showcasing the LSTM model’s capability to accurately forecast future positions.

### 3.8 Feature extraction

The purpose of feature extraction is to transform raw visual input into compact, informative representations that preserve the most discriminative aspects of vehicle appearance and structure. These representations serve as the foundational input for subsequent modules, including classification, matching, and decision-making tasks within the pipeline. In this research, we employ a dual-feature extraction strategy that combines the strengths of both DenseNet and SuperPoint architectures to capture complementary visual information. DenseNet and SuperPoint were jointly employed to extract both high-level semantic and low-level spatial features (Naseer and Jalal, 2024). DenseNet captures global class-relevant representations, while SuperPoint provides precise keypoint-based information, enhancing robustness in cluttered or partially visible aerial scenes. The following subsections provide detailed descriptions of each feature extraction technique.

#### 3.8.1 Feature extraction using DenseNet

The feature extraction phase leverages Densely Connected Convolutional Networks (DenseNet) to generate high-quality, discriminative representations from aerial vehicle images. The primary conceptual contribution of using DenseNet lies in its dense connectivity pattern, which improves gradient propagation, promotes feature reuse, and enhances representational richness without significantly increasing computational cost. Following object detection using YOLOv11, each vehicle is cropped from the original aerial frame and resized to a fixed resolution suitable for DenseNet input (Iandola et al., 2014). These vehicle image patches are then passed through the DenseNet architecture, where feature maps are progressively refined through dense blocks and transition layers. Within each dense block, every convolutional layer receives as input the concatenation of all preceding feature maps, enabling efficient multiscale feature aggregation and mitigating the vanishing gradient problem common in deep networks (Waqas and Jalal, 2024a,b). Formally Let $x_{0}$ be the initial input to a dense block the output of the I-th Layer in the block $x_{1}$ is computed as Equation 20:


(20) $$x_{1}=H_{1}(x_{0},x_{1},\ldots \ldots x_{1-1})$$

**Figure 9:**
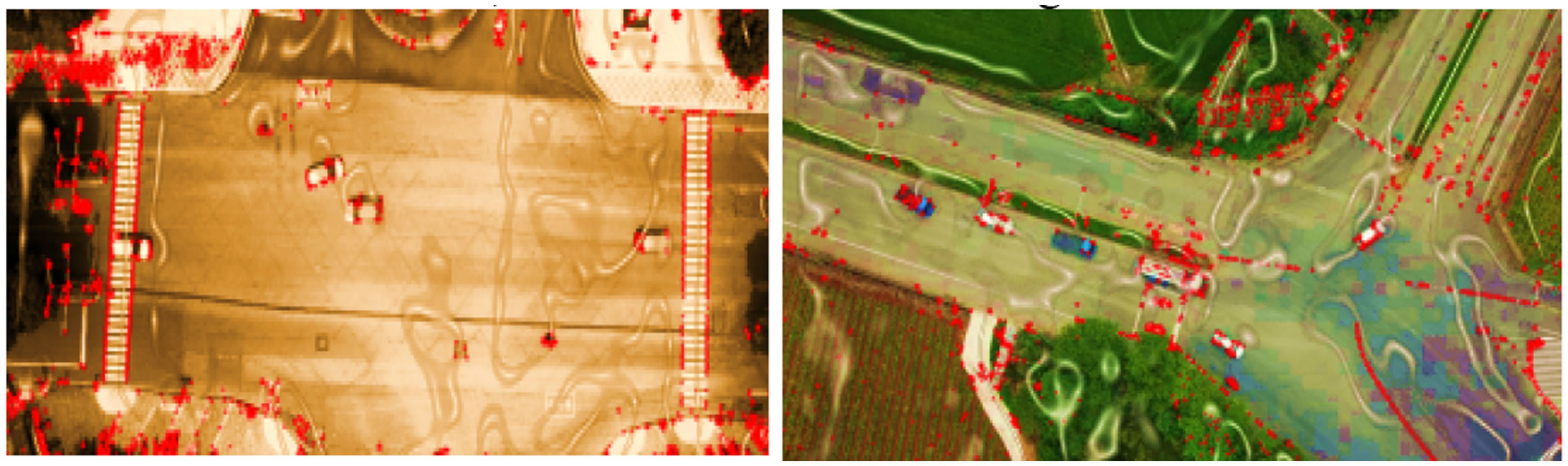
SuperPoint keypoint detection results show robust local feature extraction.

Where $H_{1}$ (.) represents a composite function of batch normalization, ReLU activation, and convolution, and [·] denotes the concatenation operation. This formulation ensures that each layer has direct access to gradients from both shallow and deep layers, resulting in more robust and diverse features for downstream tasks such as classification. The transition layers between dense blocks perform dimensionality reduction via 1 × 1 convolutions and pooling operations, allowing the network to maintain computational efficiency while preserving essential spatial information. The output feature maps encode fine-grained structural cues and global context simultaneously, making them ideal for tasks requiring high-resolution semantic detail, such as classification and behavior analysis. Equation 20 describes the core transformation mechanism that enables DenseNet to extract deep, multiscale features from aerial vehicle images. Figure 9 illustrates the output result generated by DenseNet when applied to UAVs imagery reveling its effectiveness in isolating fine grained characteristics such as contours, textures and structural edges.

#### 3.8.2 Feature extraction using SuperPoint

To complement the global semantic features extracted by DenseNet, we incorporate SuperPoint, a self-supervised convolutional neural network specifically designed for keypoint detection and descriptor extraction (Petrakis, 2023). The main conceptual contribution of integrating SuperPoint lies in its ability to identify stable, repeatable key points and generate robust local descriptors, which are particularly valuable in aerial imagery where viewpoint changes, scale variations, and partial occlusions are common (Waqas and Jalal, 2024a,b). SuperPoint operates in two stages: the interest point detector and the descriptor head. Given a grayscale image of a detected vehicle the interest point detector first outputs a heatmap identifying salient keypoints that are invariant to transformations (Mahammed et al., 2023). These points are selected based on local maxima and a predefined confidence threshold. Then, the descriptor head computes a compact 256-dimensional descriptor vector for each detected keypoint, encoding local geometric and textural information. Mathematically, let $I\in R^{H*W}$ be the grayscale input image of a vehicle. SuperPoint first produces a keypoint probability map $P\in R^{H*W}$ Such that;


(21) $$P(x,y)=\sigma (H_{d}(I))$$

**Figure 10:**
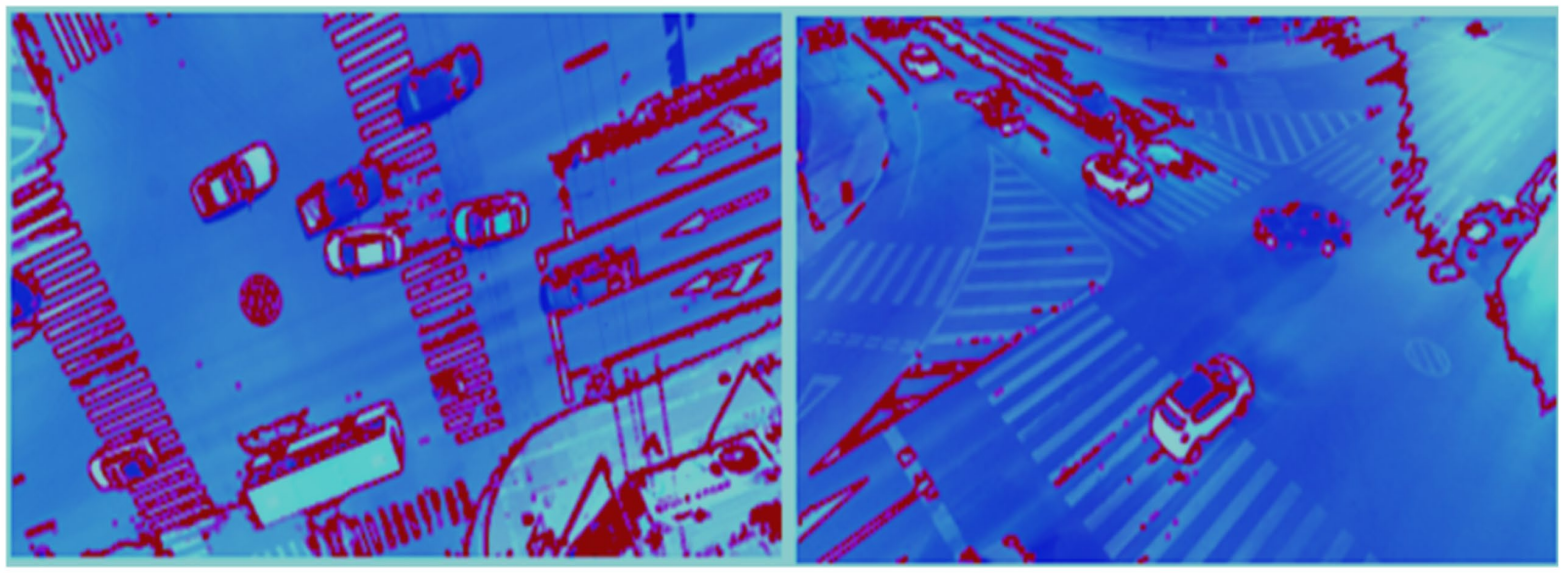
SuperPoint keypoint detection results show robust local feature extraction on vehicles.

Where $H_{d}(.)$ represents the interest point detection head and σ denotes the softmax activation applied spatially to normalize the probability distribution across the image. For each selected keypoint $(\;x_{i},y_{i}$ ) the descriptor vector $D_{i}\in R^{256}$ is computed as


(22) $$D_{i}=H_{s}\;(I,x_{i},y_{i})$$

where $H_{s}(.)$ is the descriptor head that maps local patches around the keypoint to a high-dimensional descriptor space. This local approach gives additional details to what DenseNet provides, so the system can more accurately perform fine-vehicle classifications and match places in space. Furthermore, the descriptors can be efficiently matched with other images or videos by using metrics such as cosine similarity or L2 distance which allows them to be recognized under changing conditions. SuperPoint operations for making keypoint maps and their descriptors are detailed in Equations 21, 22. Figure 10 demonstrates the SuperPoint method by highlighting different local features in aerial images.

### 3.9 Feature optimization using AutoEncoder

To enhance the quality and utility of features extracted from DenseNet and SuperPoint, a feature optimization stage is employed using a deep AutoEncoder architecture (Naseer and Ahmad, 2024). An AutoEncoder was applied after feature extraction to perform dimensionality reduction and noise suppression. It compresses the combined DenseNet and SuperPoint features into a compact latent representation, ensuring that only the most informative patterns are retained for the final classification stage. The conceptual benefit of this step lies in its ability to refine high-dimensional feature vectors by eliminating noise, reducing redundancy, and preserving only the most discriminative information (Han et al., 2018). This improves the performance of the downstream classification module while reducing computational overhead. The input to the AutoEncoder consists of concatenated feature vectors derived from the DenseNet and SuperPoint modules. Let $F_{d}\in R^{m}$ denote the DenseNet feature vector and $F_{s}\in R^{n}$ the SuperPoint descriptor aggregation. The combined input vector is given by. This concatenation is defined in Equation 23:


(23) $$F=[F_{d};F_{s}]\in R^{m+n}$$

This joint representation F is then passed to the encoder part of the AutoEncoder, which compresses it into a low-dimensional latent representation $z\in R^{k}$ , where k≪m+n . The encoder learns a nonlinear transformation E (.) such that. This transformation is represented by Equation 24:


(24) $$z=E(F)=\phi (W_{e}\;F+b_{e}\;)$$

where $w_{e}$ and $b_{c}$ are the encoder weights and biases, and *ϕ* denotes the activation function (e.g., ReLU). The decoder reconstructs the original input from z using a symmetric mapping. This decoding process is formulated in Equation 25:


(25) $$F=D(z)=\phi (W_{d}\;z+b_{d}\;)\;$$

where $w_{d}$ and $b_{d}$ are the decoder parameters. The AutoEncoder is trained to minimize the reconstruction loss, typically the mean squared error (MSE) between the original and reconstructed feature vectors. The reconstruction loss is defined in Equation 26.


(26) $$L_{\mathrm{rec}}=F-F\frac{2}{2}\;$$

**Figure 11:**
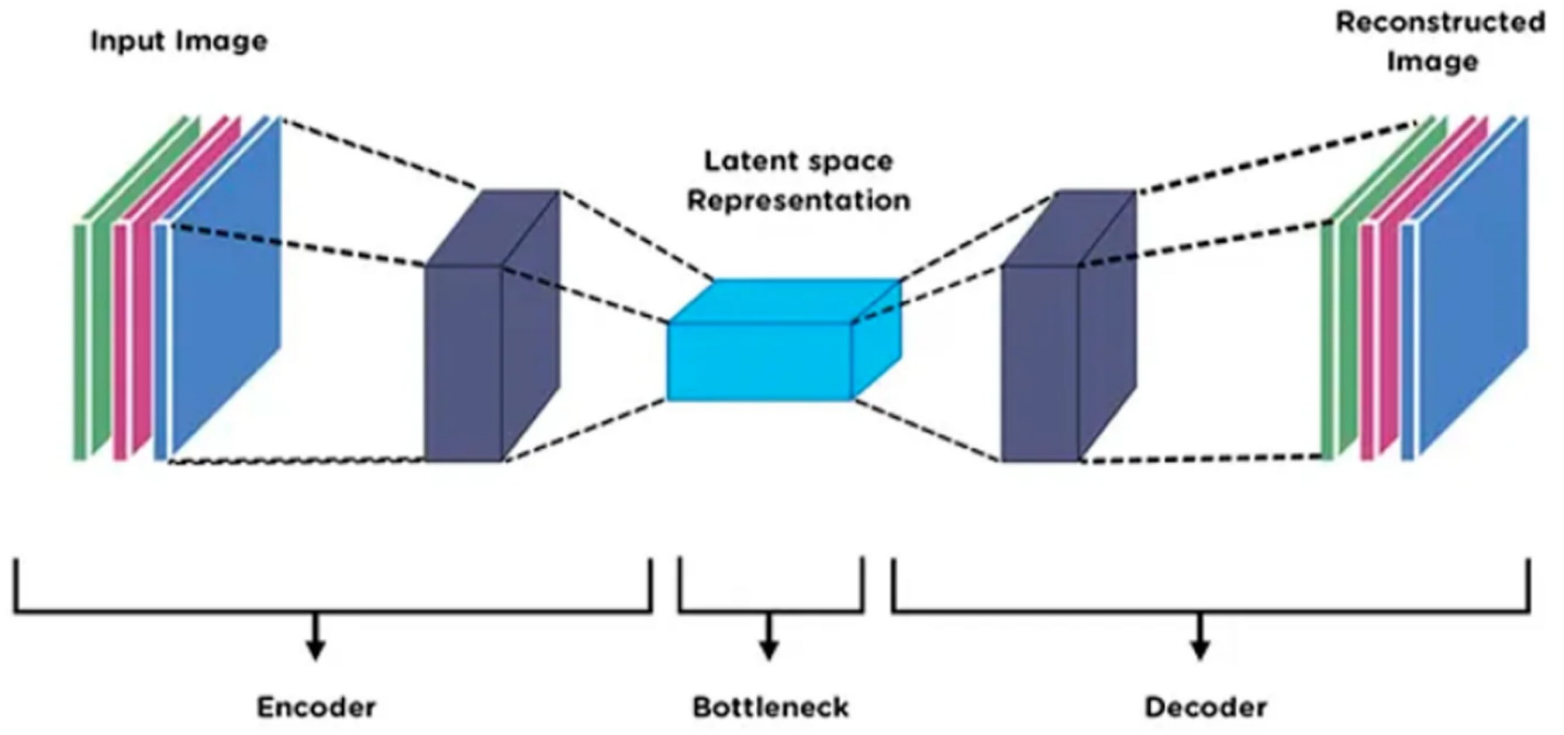
AutoEncoder framework with refined feature vectors from DenseNet and SuperPoint.

This latent representation z serves as the optimized feature vector fed into the classification stage. It retains only the most salient and discriminative attributes of the vehicle images, improving generalization, especially in complex aerial environments with high intra-class variability. The AutoEncoder enforces a compact and structured feature space, which enhances classification accuracy and efficiency. Figure 11 illustrates the architecture of the AutoEncoder.

### 3.10 Vehicle classification using vision transformer (ViT)

The final stage in the proposed deep learning–based aerial vehicle analysis pipeline is vehicle classification, which leverages the Vision Transformer (ViT) architecture. ViT was chosen for its ability to capture global contextual relationships across the entire feature map using attention mechanisms, improving the interpretability and accuracy of final vehicle classification (Waqas M. and Ahmad, 2024). The primary conceptual contribution of employing ViT lies in its attention-based modeling, which enables the network to capture global contextual relationships across image patches, resulting in improved interpretability and discriminative power, especially valuable in aerial views where vehicle appearances may vary due to occlusion, scale, or orientation (Dong et al., 2024). In this stage, the optimized feature vector $z\in R^{k}$ , obtained from the AutoEncoder, is reshaped and embedded into a fixed-length sequence to serve as input tokens for the transformer encoder. Each token is processed in conjunction with a learnable positional embedding to retain spatial ordering (Naseer and Jalal, 2025a,b; Waqas A. and Ahmad, 2024). The ViT encoder consists of multiple layers of multi-head self-attention (MHSA) and feedforward neural networks, enabling the model to focus on relevant feature interactions and suppress irrelevant noise. Mathematically, the input sequence $X\in R^{N*D}$ is computed as:


(27) $$X=[z_{1}\;E;z_{2}E;\ldots \ldots .;z_{N}E]+P$$

This sequence embedding is defined in Equation 27. Where $\mathrm{zi}\in R^{k}$ are the segmented features, $E\in R^{k*d}$ is the linear projection matrix, and $P\in R^{n*d}$ is the positional embedding. Each encoder block applies MHSA:


(28) $$\text{MHSA}(Q,K,V)=\text{Concat}\;(h_{1},,,,,,,h_{H})W$$

The MHSA operation is defined in Equation 28. Where $h_{i}$ = Attention $\left(Q_{i},K_{i},V_{i}\right)$ and Q, K, V are projections of X the attention weight are computed as;


(29) $$\text{Attention}\;(Q,K,V)=\text{Softmax}\;(\frac{QK^{t}}{\sqrt{\;d_{k}}})V$$

**Figure 12:**
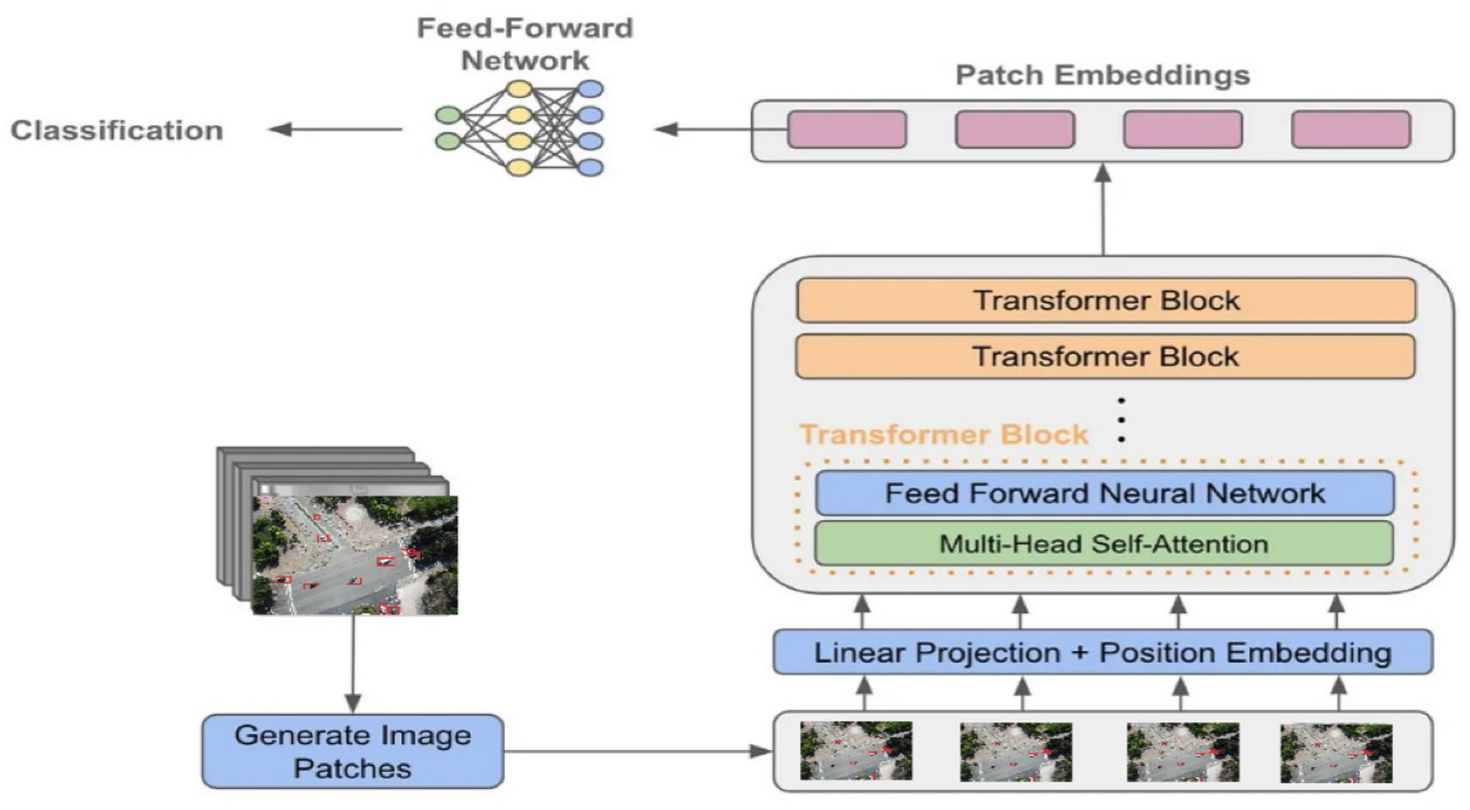
Architecture of the Vision Transformer (ViT) used for vehicle classification.

The attention weights are computed as shown in Equation 29. After processing through several attention layers, the class token is passed to a classification head, typically a fully connected layer, to predict the vehicle type (e.g., car, truck, bus, or van). This final decision leverages the refined and contextually enriched feature representation from earlier stages, allowing for accurate classification even in cluttered and dynamically changing aerial environments (Waqas and Jalal, 2025). The above equations describe the embedding, attention, and classification process within the ViT framework. Figure 12 illustrates the Vision Transformer architecture used in our proposed pipeline.

## 4 Experimental setup and datasets

The experiments were done on a high-performance computer that was organized for deep learning tasks. With Windows 11 installed, the machine houses an Intel Core i9-13900K processor running at 3.70 GHz and 24 cores, 64 GB of DDR5 RAM, and an NVIDIA RTX A6000 graphic card that is equipped with 48 GB of memory and 10,752 CUDA cores. Because of these configurations, the pipeline could process the needed models quickly and parallely which included HRNet, YOLOv11 and Vision Transformers for different image processing jobs. Python 3.10 and PyTorch 2.1 were used for the development of the framework. CUDA 12.2 was in charge of GPU acceleration and the use of NumPy, OpenCV and SciPy libraries helped with data preprocessing, augmentation, and displaying images. A total of 80% of the data was used for training, while the testing was done using the remaining 20% to ensure equality and wider usefulness. Both sets were designed to include many types of scenes and vehicles to check how powerful the system would be when facing changes in scale, the number of objects around, and weather visibility.

### 4.1 Datasets

For a thorough evaluation of the pipeline, we applied two benchmark datasets called AU-AIR and Roundabout. The sets of data were chosen so that performance assessment could handle a variety of environmental, traffic, and structure circumstances.

#### 4.1.1 AU-AIR

The dataset supplied by AU-AIR is impressive because it was collected with different types of UAVs and we found that useful due to the various altitudes and angles of the captured images taken at various times and weather conditions (Bozcan and Kayacan, 2020). The AU-AIR dataset contains five primary object classes: car, truck, bus, motorbike, and bicycle, with cars being the dominant category. All images were resized to 416 × 416 pixels and normalized to a [0,1] range before training. To improve generalization, we applied data augmentation techniques, including horizontal flipping, random rotation, and brightness adjustment. Although AU-AIR is relatively balanced, we applied mild class weighting in the loss function to ensure consistent learning performance across all vehicle types. Because cars, trucks, buses and motorbikes were part of the data, there was a lot of variation that could challenge both fine class estimation and the model’s ability to cope with many types of images.

#### 4.1.2 Roundabout dataset

**Figure 13:**
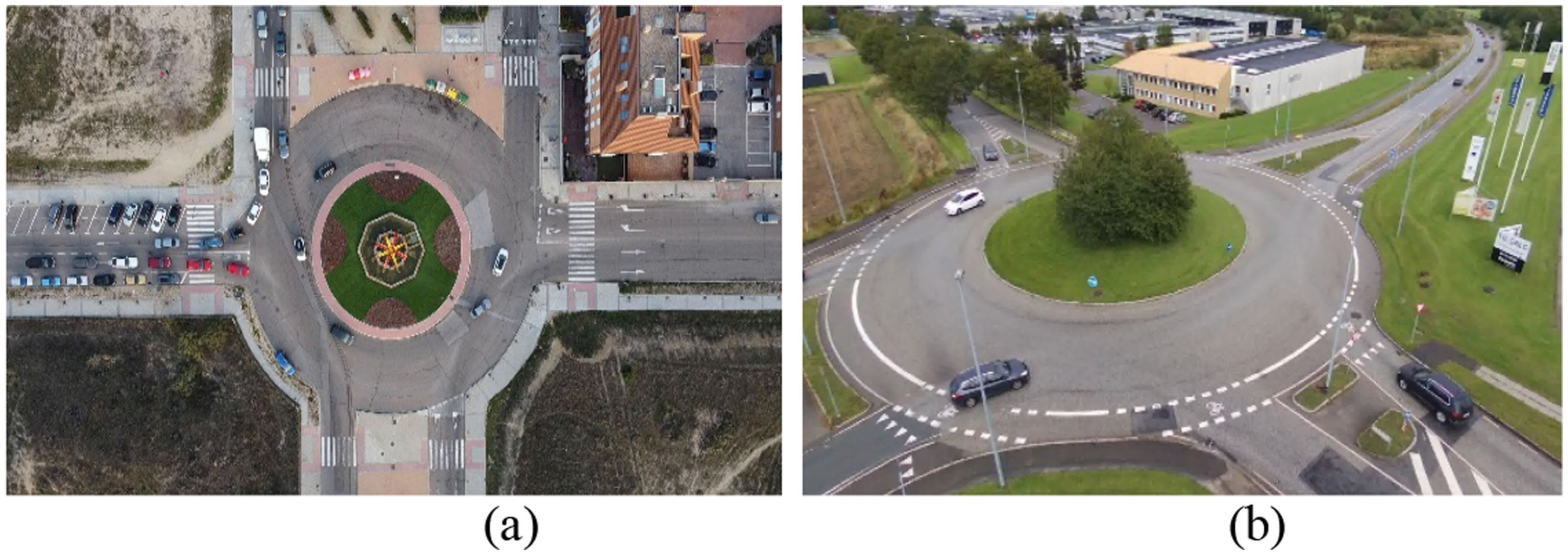
Sample images from the VAID **(a)** and AU-AIR **(b)** dataset.

Using the Roundabout data, we perform detailed studies on aerial vehicles, mainly focusing on complicated traffic situations (Puertas et al., 2022). It uses images shot by UAVs and focuses on capturing roundabouts, where heavy, erratic traffic and overlappings of vehicles often make it hard to see everything in one shot. Videos are captured using excellent resolution and include a lot of information about the positions and movements of traffic. The Roundabout dataset is imbalanced, with most samples being cars, and trucks, Sedan, Cement Truck, Trailer and Bus. Images were resized to 416 × 416 and normalized. Augmentations matched AU-AIR. To address the imbalance, we used weighted loss and balanced mini-batch sampling. From what we have seen, it serves as a standard and provides a realistic insight into the challenges of working with autonomous aerial systems. Figure 13 shows the samples images of Roundabout dataset and VAID dataset.

### 4.2 Model evaluation

To evaluate the performance of our framework, we used two recognized benchmark datasets named AU-AIR and Roundabout. The proposed framework was thoroughly tested using the AU-AIR and Roundabout benchmark data to check how well it coped with various aerial transportation scenarios. To verify the results and limit the effect of anything occurring by chance, each experiment was carried out five times on its own Hanzla and Jalal (2025). The data analysis is reliable since the averages give a steady and statistically valid set of numbers. Table 1 shows the evaluation for every core module along with the precision, recall and F1-score. These findings indicate that the pipeline performs well even in difficult circumstances, like when things are obscured, moving fast or lighting varies which proves how suitable and robust it is for real-world situations involving UAVs. These consistent and robust results across both the AU-AIR and Roundabout datasets, which vary in camera type, environment, and traffic complexity, confirm the system’s cross-platform scalability and adaptability in diverse UAV applications.

**Table 1:** Precision, recall, and F1-score for the detection algorithm.

Datasets	Precision	Recall	F1-score
AU-AIR	97.8	95.0	96.6
Roundabout	96.9	94.4	95.5

Table 2 presents the confusion matrix for vehicle classification results on the AU-AIR dataset, showcasing the model’s ability to distinguish between various vehicle categories under challenging aerial conditions. Table 3 provides detection performance metrics, including accuracy, precision, recall, and F1-score for the same dataset, highlighting the robustness of the YOLOv11-based detection module. In parallel, Table 4 shows the classification matrix for the Roundabout dataset, while Table 5 reports its detection metrics, further validating the pipeline’s adaptability across different scene layouts and traffic densities Hanzla and Jalal (2025). Table 6 compares the classification accuracy of the Vision Transformer with other baseline models, illustrating the superiority of attention-based architectures in aerial imagery. Table 7 evaluates tracking performance using Deep SORT across both datasets, emphasizing consistent identity preservation even under occlusion and motion variation. Finally, in Table 8 we compare our classification of AU-AIR and Roundabout datasets, with those of other authors who have use these datasets for classification, confirming the proposed framework effectiveness, cross-dataset generalizability, and practical applicability in intelligent aerial traffic monitoring systems.

**Table 2:** Confusion matrix for vehicle classification on AU-AIR dataset.

Classes	C	Tru	B	Cy	V	MB	Tra
C	99	0	0	0	0	0	1
Tru	1	98	0	0	0	1	0
B	0	0	98	0	1	1	0
Cy	0	0	0	98	0	1	1
V	0	0	0	1	98	0	1
MB	1	0	0	0	0	99	0
Trs	1	0	0	0	0	0	99
Mean: 98.4							

Mn = Minibus, TR = Truck, PT = Pickup Truck, B=Bus, SD=Sedan, C=Car, CT = Cement Truck, Tra = Trailer.

**Table 3:** Detection accuracy, precision, recall, and F1-score evaluation of AU-AIR dataset.

Classes	Precision	Recall	F1-score
Mn	98.2	94.7	96.4
TR	97.5	95.5	96.5
PT	97.6	95.2	96.4
B	98.0	94.8	96.3
SD	97.9	95.0	96.4
C	97.8	95.0	96.3
CT	97.9	95.1	96.4
Tra	98.5	95.1	96.9
Mean	97.8	95.0	96.6

Mn = Minibus, TR = Truck, PT = Pickup Truck, B=Bus, SD=Sedan, C=Car, CT = Cement Truck, Tra = Trailer.

**Table 4:** Confusion matrix for vehicle classification over the roundabout dataset.

Classes	Mn	Tr	PT	B	SD	C	CT
Mn	98	1	0	0	0	1	0
TR	0	98	1	0	0	1	0
PT	0	0	98	1	1	1	0
B	0	1	0	97	97	0	1
SD	0	0	1	1	1	0	1
C	1	0	1	0	0	98	0
CT	0	0	0	1	1	0	98
Mean: 97.7							

Mn = Minibus, TR = Truck, PT = Pickup Truck, B=Bus, SD=Sedan, C=Car, CT = Cement Truck, Tra = Trailer.

**Table 5:** Detection accuracy, precision, recall, and F1-score evaluation of roundabout dataset.

Classes	Precision	Recall	F1-score
Mn	97.5	94.0	95.7
TR	97.0	93.5	95.5
PT	96.5	94.5	95.0
B	96.0	94.0	95.1
SD	97.5	93.0	95.1
C	96.0	94.0	95.0
CT	97.0	97.0	95.4
Tra	97.5	95.0	96.2
Mean	96.9	94.4	95.5

Mn = Minibus, TR = Truck, PT = Pickup Truck, B=Bus, SD=Sedan, C=Car, CT = Cement Truck, Tra = Trailer.

**Table 6:** Comparison of model detection rate with other state-of-the-art methods.

Datasets	Models	Precision
AU-AIR	Yolov7	87.0
	EfficientDet	91.0
	MSER + EdgeBoxes	84.0
	Our method	97.8
Roundabout	ATSS Detector	88.0
	NDFT	91.0
	Blob detection	73.0
	Our method	96.9

**Table 7:** Comparison of model tracking rate with other state-of-the-art methods.

Datasets	Models	Precision
AU-AIR	Kalman filter + HOG	91.0
	SiamRPN++	93.0
	Particle Filter	88.0
	Our method	96.5
Roundabout	ECO tracker	85.2
	FairMOT	77.0
	Template matching	89.1
	Our method	94.4

**Table 8:** Classification comparison with other state-of-the-art models.

Method	AU-AIR	Roundabout
Tas et al.	94.5%	95.6%
Kumar et al.	92.1%	96.2%
L. Du et al.	85.7%	–
H. Zhang et al.	–	87.4%
Y. Wang et al.	94.9%	–
Proposed method	98.4%	97.7%

### 4.3 Ablation study and efficiency analysis

To evaluate the contribution of each component within the proposed framework, we conducted an ablation study by selectively removing or replacing individual modules. As summarized in Table 9, removing RetinexNet resulted in noticeable degradation in detection and tracking accuracy, confirming its importance in enhancing low-visibility UAV imagery. Replacing CSRNet with a basic CountCNN caused a drop in classification precision due to reduced accuracy in vehicle density mapping. Excluding the AutoEncoder slightly affected classification accuracy, while removing ViT in favor of DenseNet-only classification led to a more significant performance drop, underscoring the advantage of attention-based global feature modeling in ViT.

**Table 9:** Effect of removing modules on overall system performance across three tasks.

System configuration	Detection accuracy (%)	Tracking accuracy (%)	Classification accuracy (%)
Full Framework (All Modules)	97.8	96.5	98.4
Without RetinexNet (No Enhancement)	94.2	93.1	95.6
Replacing CSRNet with CountCNN	97.8	96.5	96.3
Without AutoEncoder	97.8	96.5	96.9
Without ViT (using DenseNet only)	97.8	96.5	95.2

In addition to performance impact, we analyzed the runtime efficiency and hardware resource requirements of the complete system. This is especially critical for UAV deployment, where real-time performance is often constrained by limited onboard processing. As summarized in Table 10, the system achieves an average inference time of 54 ms per frame, equating to approximately 18.5 FPS. The peak memory usage during inference was measured at 5.1 GB on an NVIDIA RTX 2080 Ti GPU. The full framework includes approximately 62 million trainable parameters, with a total model size of 250 MB, making it computationally efficient and scalable to edge GPU platforms suitable for UAVs.

**Table 10:** Runtime performance, memory usage, and model complexity of the proposed framework.

Metric	Value
Inference Time (per frame)	54 ms
Effective Speed	~18.5 FPS
GPU Used	NVIDIA RTX 2080 Ti
Peak Memory Usage (Inference)	5.1 GB
Total Model Size	~250 MB
Trainable Parameters	~90 million

## 5 Limitation of proposed framework

While the proposed aerial vehicle analysis pipeline achieves strong performance across multiple tasks, there remain areas that offer valuable opportunities for further enhancement. Environmental variations such as changing lighting, shadows, and weather conditions naturally introduce complexities in aerial image quality, which may affect segmentation and detection accuracy however, these challenges also open avenues for developing more robust preprocessing and adaptive learning methods. Dense and occluded traffic scenes present intricate scenarios where vehicle boundary precision and identity tracking can be refined, suggesting potential for improved multi-scale feature modeling and advanced attention mechanisms. Although LSTM-based trajectory prediction effectively models temporal dynamics, exploring hybrid or more sophisticated temporal architectures could better capture complex, nonlinear vehicle motions. The computational demands of integrating multiple deep learning models encourage the pursuit of more efficient architectures and model compression techniques to maintain real-time feasibility on UAV hardware. Additionally, expanding dataset diversity and annotation quality remains a priority to further enhance model generalization, presenting exciting prospects for leveraging synthetic data generation and active learning. Overall, these considerations highlight the ongoing potential to strengthen and extend the pipeline’s capabilities without detracting from its foundational achievements. Furthermore, as the current evaluation is limited to two datasets, the generalizability of the framework to unseen aerial environments (e.g., rural, coastal, or emergency settings) remains to be fully validated. Additionally, the system depends on several pre-trained modules, which may require domain adaptation or fine-tuning when applied to datasets with significantly different characteristics, such as varying UAV altitudes, sensor types, or camera angles.

## 6 Conclusion and future work

The study suggests using a complete deep learning framework for examining aerial vehicles, tackling all types of obstacles found in UAV-based traffic perception such as obscurities, changing sizes, moving vehicles and complex environments. With the help of advanced models such as RetinexNet for preprocessing, HRNet for high-resolution segmentation, YOLOv11 for effective detection, Deep SORT for keeping track of vehicles, CSRNet for density-based vehicle counting, LSTM for predicting trajectories and DenseNet, SuperPoint for feature extraction and autoencoder for future optimization then ViT for vehicles classification. It gives a complete solution that is accurate and reliable for aerial images. The outcome clearly proves that the AU-AIR and Roundabout datasets give superior results for detection, tracking and classification, as well as indicate that the sensor performs dependably in traffic monitoring and autonomous navigation systems. Our future work will focus on optimizing the framework for real-time deployment on low-power UAV hardware through model compression and architectural simplification. We also plan to extend the evaluation to additional aerial datasets featuring more varied geographic, environmental, and traffic conditions. To improve generalizability across diverse scenes with limited annotations, we will explore lightweight domain adaptation techniques. In addition, improving low-light and night-time performance using enhanced data preprocessing or fine-tuned models remains a practical next step. These incremental improvements are expected to further enhance the system’s robustness and deployability in real-world UAV traffic monitoring scenarios.

## Data Availability

Publicly available datasets were analyzed in this study. This data can be found at: https://www.kaggle.com/datasets/javiersanchezsoriano/roundabout-aerial-images-for-vehicle-detection.
